# Improving Lung Cancer Risk Prediction Using Machine Learning: A Comparative Analysis of Stacking Models and Traditional Approaches

**DOI:** 10.3390/cancers17101651

**Published:** 2025-05-13

**Authors:** Huakang Tu, Yunfeng Zhao, Jiameng Cui, Wanzhu Lu, Gege Sun, Xiaohang Xu, Qingfeng Hu, Kejia Hu, Ming Wu, Xifeng Wu

**Affiliations:** 1 Center of Clinical Big Data and Analytics of the Second Affiliated Hospital and School of Public Health, Zhejiang University School of Medicine, Hangzhou 310058, China; yunfengz@zju.edu.cn (Y.Z.); 22218259@zju.edu.cn (J.C.); 22218817@zju.edu.cn (W.L.); 22218811@zju.edu.cn (G.S.); 12018344@zju.edu.cn (X.X.); hu_qingfeng@zju.edu.cn (Q.H.); kejiahu@zju.edu.cn (K.H.); 2 Department of Thoracic Surgery, The Second Affiliated Hospital, Zhejiang University School of Medicine, 88 Jiefang Rd., Hangzhou 310009, China; iwuming22@zju.edu.cn; 3 National Institute for Data Science in Health and Medicine, Zhejiang University, Hangzhou 310058, China; 4 Zhejiang Key Laboratory of Intelligent Preventive Medicine, Hangzhou 310058, China

**Keywords:** machine learning, prediction, lung cancer, stacking models, artificial intelligence

## Abstract

Background: Lung cancer is a leading cause of cancer-related mortality worldwide, often diagnosed in advanced stages, making early detection critical. This study aimed to evaluate the performance of various machine learning models in predicting lung cancer risk based on epidemiological questionnaires, comparing them with traditional logistic regression models. Methods: A retrospective case–control study was conducted using data from 5421 lung cancer cases and 10,831 matched controls. The dataset included a wide range of demographic, clinical, and behavioral risk factors from epidemiological questionnaires. We developed and compared multiple machine learning algorithms, including LightGBM and stacking ensemble models, alongside logistic regression for predicting lung cancer risk. Model performance was evaluated using accuracy, area under the curve (AUC), and recall. Results: The stacking model outperformed traditional logistic regression, achieving an AUC of 0.887 (0.870–0.903) compared to 0.858 (0.839–0.878) for logistic regression. LightGBM also performed well, with an AUC of 0.884 (0.867–0.901). The stacking model achieved an accuracy of 81.2%, with a recall of 0.755, higher than the logistic regression model’s accuracy of 79.4%. Compared to classical lung cancer prediction models (LLP and PLCO), the logistic regression and ML models improved AUC by 12% to 27%. Conclusions: Integrating machine learning models into lung cancer screening programs can significantly enhance early detection efforts. Machine learning approaches, such as LightGBM and stacking, offer improved accuracy and predictive power over traditional models. However, efforts to enhance model interpretability through explainable AI techniques are necessary for broader clinical adoption.

## Simple Summary

Machine learning models could demonstrate superior predictive performance for lung cancer risk compared to traditional logistic regression and classical prediction models (Liverpool Lung Project model, and the Prostate, Lung, Colorectal, and Ovarian Cancer Screening model) utilizing data from epidemiological questionnaires. In a retrospective case–control study of 5421 lung cancer cases and 10,831 matched controls, the stacking model achieved an AUC of 0.887 and an accuracy of 81.2%, outperforming logistic regression, which had an AUC of 0.858 and an accuracy of 79.4%. Both machine learning models significantly improved AUC by 12% to 27% compared to classical lung cancer prediction models. Integrating machine learning models into lung cancer screening programs can substantially enhance early detection efforts, necessitating further exploration of explainable AI techniques for clinical applicability.

## 1. Introduction

Lung cancer is one of the most prevalent and lethal cancers worldwide, representing a significant public health challenge [1]. Despite advancements in diagnostic and therapeutic modalities, the majority of lung cancer cases are diagnosed at advanced stages, resulting in a poor prognosis and a 5-year survival rate of less than 20% in most populations. Globally in 2022, there were over 2.4 million new cases of lung cancer and 1.8 million deaths attributed to the disease, underscoring its devastating burden on healthcare systems and societies [2]. Early detection of lung cancer through risk prediction tools could significantly reduce mortality by enabling timely interventions and improving prognosis. Developing early cancer risk prediction tools can help detect cancer early and reduce cancer mortality rate. Several lung cancer risk prediction models have been developed to screen for high-risk individuals, integrating epidemiological, clinical, and behavioral risk factors. Across these models, age, smoking history (including smoking status, duration, cigarettes per day, pack-years, and years since quitting), and family history are consistently recognized as primary determinants of lung cancer risk. Smoking, in particular, remains the single most significant risk factor, accounting for approximately 80% of all lung cancer cases. Many studies are limited to small sample sizes, or involve only specific high-risk populations (e.g., smokers or occupationally exposed populations), limiting the ability of the model to generalize. However, lung cancer in never-smokers, which constitutes 10–25% of cases, highlights the importance of incorporating additional risk factors into prediction models. Additionally, some models also incorporate factors such as sex, body mass index (BMI), chronic obstructive pulmonary disease (COPD), and occupational exposure, particularly focusing on the hazards of asbestos and dust exposure [3,4,5,6,7,8,9,10,11,12,13,14,15,16,17].

In recent years, the rise of machine learning (ML) has provided transformative tools for medical risk prediction, offering enhanced accuracy and predictive power. Traditional statistical methods such as logistic regression, though robust, may not fully capture the complex interactions between risk factors. Advanced machine learning algorithms, including random forest, extreme gradient boosting (XGBoost), and multilayer perceptron (MLP), have demonstrated superior performance in modeling nonlinear relationships and handling high-dimensional datasets [18,19,20]. Furthermore, ensemble learning techniques, particularly stacking, have shown promise in improving prediction accuracy by combining the strengths of multiple base models. Unlike bagging or boosting methods that focus on refining predictions through iterations, stacking leverages heterogeneous algorithms to create a meta-model, thus addressing variability and improving overall robustness [21,22]. However, there remains uncertainty regarding the optimal machine learning approach for early lung cancer risk prediction. While individual models have shown remarkable accuracy, the application of ensemble methods, such as stacking, in real-world epidemiological datasets has yet to be fully explored. This gap is particularly evident in populations with varying smoking behaviors, such as never-smokers, current smokers, and former smokers, where risk profiles and associated factors differ substantially. Tailoring predictive models to these subgroups is crucial for improving discriminatory performance and enhancing the practical utility of such tools in diverse populations.

To address these gaps, this study leverages epidemiological questionnaire data focused to develop and compare machine learning models like LightGBM and stacking ensemble approaches on a large-scale sample size from Zhejiang, China, to systematically evaluate the performance of different machine learning methods for early lung cancer risk prediction. The study benchmarks logistic regression, random forest, XGBoost, MLP, and stacking models, assessing their discriminatory ability across all participants as well as stratified smoking subgroups. The stacking model demonstrated superior discriminatory capabilities, achieving AUCs of 0.887, 0.901, 0.837, and 0.814 for the overall dataset, never-smokers, current smokers, and former smokers, respectively.

## 2. Materials and Methods

### 2.1. Study Population and Epidemiological Data

A total of 5421 lung cancer patients with confirmed diagnosis of non-small cell lung cancer (NSCLC) were recruited from Second Affiliated Hospital of Zhejiang University (SAH) between 2020 and 2023. The healthy control subjects were recruited at Lanxi and SAH from 2020 to 2023. The control subjects from Lanxi and SAH visited the general practice department for annual checkups. A total of 10,831 control subjects were matched to the lung cancer patients by age, sex, and smoking status. All subjects enrolled in the study were asked to sign an informed consent form (ethics approval numbers: 2019LSYD338, 2022LSYD0805), approved by the Institutional Review Board of SAH, before being interviewed, and an epidemiological questionnaire was administered by trained interviewers.

Detailed information was collected through an epidemiological questionnaire, including demographic characteristics, smoking history, drinking history, diet habits, sleeping quality, exposures at work and medical history. Extensive information about smoking was gathered, including smoking status, exposure to second-hand smoke, ever living with smokers for more than half a year and the number of cigarettes smoked per day. A never-smoker was someone who had never smoked or had smoked fewer than 100 cigarettes in their lifetime [23]. A former smoker was defined as an individual who had smoked more than 100 cigarettes and had successfully quit smoking. An individual who had smoked more than 100 cigarettes but had not quit smoking (i.e., was still smoking) was classified as a current smoker.

All participants were asked if they had been diagnosed with non-malignant lung disease by physicians before the interview, such as chronic bronchitis, asthma, chronic obstructive pulmonary disease, tuberculosis and pulmonary nodule. Self-reported cancer diagnoses in parents, siblings and children were collected and primary sites were recorded. Information on self-reported work exposure (at least 5 years) to heavy metal, diesel, coal dust and asbestos was collected. Physical activity was divided into four categories: inactive (≤3.75 metabolic equivalent (MET) hours/week), low active (3.75–7.5 MET hours/week), medium active (7.5–16.5 MET hours/week) and high active (≥16.5 MET hours/week). Metabolic equivalent hours per week was defined as the product of intensity, frequency of exercise and duration of physical activity [24]. Sleeping quality was assessed by the frequency of participants experiencing conditions, such as difficulty in falling asleep, coughing and feeling pain during sleep in the past month.

### 2.2. Data Preprocessing and Imputation

We randomly partitioned 80%, 10% and 10% of participants into training, validation and test datasets. Before data imputation, we kept features with proportion of missing values less than 25%, respectively, and 32 features left for analysis. Then, we imputed the missing values of training, validation and test data separately with R-package missForest (version 1.5). The advantage of missForest is its ability to handle mixed-type data, complex interactions and nonlinear relationship [25]. For categorical variables with more than two levels, one-hot encoding is applied to ensure its integration into a numerical machine learning framework [26]. Then, Z-score normalization is performed across variables to ensure comparable feature scales and improve model convergence [27]. The variable z was calculated as z = (x − μ)/s, where μ was the mean of the variable and s was the standard deviation of the variable in the training dataset.

### 2.3. Model Development and Evaluation

We trained eight traditional machine learning (ML) models, including regularized logistic regression (LogiR), random forest (RF), light gradient-boosting machine (lightGBM), extra trees (ET), extreme gradient boosting (XGBoost), adaptive boosting (AdaBoost), gradient boosting decision tree (GBDT) and support vector machine (SVM) to predict the risk of lung cancer with all variables, together with a deep learning (DL) model, which is the multilayer perceptron. In the model training process, we fitted nine baseline models with five-fold cross validation to fine-tune the model parameters, and a random search was conducted to find the best combination of hyper-parameters by using the RandomizedSearchCV function in Scikit-learn library (version 1.4.2). To construct the stacking model, we first computed the predicted probability of the aforementioned nine base models and evaluated model performance on the test dataset. Then, we defined stacking model using the StackingClassifier from sklearn.ensemble module. The “estimators” argument of the Stacking Classifier included five base models with the highest AUCs, and the “final_estimator” argument was set to logistic regression classifier. The input of the logistic regression was the predicted probability of five base models. After all ML models were fitted, the performance of the models was evaluated by sensitivity, specificity, area under the ROC curve (AUC) and calibration curve. The relative importance of the stacking model was calculated by a permutation test.

### 2.4. Statistical Analysis

For descriptive analysis, median (IQR) was used for continuous variables and count (percent) was used for categorical variables. Distributions in baseline characteristic variables between lung cancer patients and healthy subjects were evaluated by a Chi-square test for categorical variables and Wilcoxon rank sum test for continuous variables with distributions deviated from the normal. The descriptive statistics and comparison were calculated by R-package tableone (version 0.13.2). The OR and 95% confidence intervals (CIs) in univariate and multivariate logistic regression were obtained from the glm function in R-package stats (version 4.3.1). The one-hot encoding transformation was implemented by OneHotEncoder from Scikit-learn library (version 1.4.2) in Python. The ML model training and evaluation pipeline uses packages including Scikit-learn, xgboost (version 2.1.1) and lightgbm (version 4.5.0) in Python. All data processing, statistical analysis and plotting were conducted in R (version 4.3.1, RStudio, Boston, MA, USA) and Python (version 3.12.2, Python Software Foundation, Fredericksburg, VA, USA). All statistical tests were two-sided. *p* < 0.05 was regarded as statistically significant.

## 3. Results

### 3.1. Baseline Characteristics

Baseline characteristics of 5421 lung cancer patients and 10,831 healthy control subjects are summarized in Table 1. Since the lung cancer patients and healthy control subjects were matched by age, sex and smoking status, there was no statistically significant difference between the distribution of case and control groups. About 75% of subjects included in the study were never-smokers, and there were more former smokers (17%) than current smokers (8%) in both groups. More lung cancer patients (45%) were exposed to second-hand smoke than healthy subjects (28.1%), and 24.1% of lung cancer patients had lived with smokers for more than half a year, while the percentage was only 12.8% in healthy subjects. The distributions of education and income were statistically significantly different between the case and control groups. Overall, the education and income levels of lung cancer patients were slightly higher than the healthy subjects. Significant differences were observed in other epidemiological risk factors including physical activity (*p* < 0.001), drinking status (*p* < 0.001), family history of lung cancer (*p* < 0.001), and prior diagnosis of non-malignant lung disease (*p* < 0.005).

### 3.2. Model Performance

In general, the eight baseline machine learning models (LogiR, RF, LightGBM, ET, XGBoost, AdaBoost, GBDT, SVM) and one deep learning model (MLP) all achieved good performance in predicting the risk of lung cancer in the test dataset, which quantifies the models’ ability to distinguish between lung cancer cases and controls (Table 2 and Supplementary Materials Tables S1–S4) and calibration (Supplementary Materials Figure S1). For predictions in full data, the lightGBM model achieved the highest AUC of 0.884 in the test dataset, while MLP achieved fairly good prediction with an AUC of 0.877. For predictions in never-smokers, lightGBM achieved the highest AUC of 0.897 (95% CI: 0.879–0.915) in the test dataset. Furthermore, we integrated the top five baseline models to create a stacking model for boosting predictive performance. In full dataset prediction, the stacking models achieved an AUC of 0.887 (95% CI: 0.870–0.903), which exceeded the AUC of the multivariate logistic regression model by 3%, and other ML models by a range of 1–5%. In current and former smokers, the performance of the stacking model was slightly better than logistic regression models, while some ML models and the MLP model achieved lower AUCs than the logistic regression model. When compared to classical lung cancer prediction models, such as LLP and PLCO, our multivariate logistic regression model and stacking models improved AUC by a range of 12% to 27% (Table 2).

### 3.3. OR and Feature Importance

**Figure 1:**
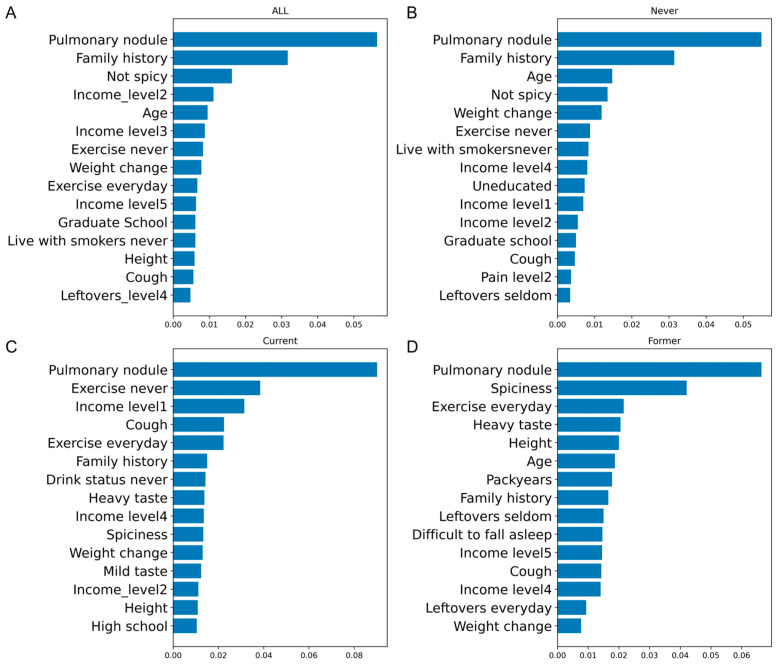
Permutation feature importance from the stacking model. (**A**) Permutation feature importance for the full dataset. (**B**) Permutation feature importance for never-smokers. (**C**) Permutation feature importance for current smokers. (**D**) Permutation feature importance for former smokers.

Variables ranked high in feature importance plots (Figure 1) also had high OR by logistic regression (Table 3). For example, pulmonary nodule ranked as the most significant variable across all feature plots, and the OR of pulmonary nodule for the full data model is 41.32 (95% CI: 31.40–54.36), 42.815 (95% CI: 30.86–59.39) for never-smokers, 98.10 (95% CI: 26.91–357.66) for current smokers and 33.75 (95% CI: 18.67–61.00) for former smokers. The family history of lung cancer was significantly associated with an increased risk of developing lung cancer, with the OR ranging from 3.22 to 6.29. Living with smokers for more than a half year also ranked high in feature importance plot of full data for never-smokers. The OR of living with smokers currently is 2.06 (95% CI: 1.82–2.34).

## 4. Discussion

In this study, we aimed to develop and compare various machine learning models with traditional regression-based methods for predicting lung cancer risk based on extensive epidemiological risk factors. Our results demonstrate that machine learning algorithms, particularly the stacking model, provided superior performance compared to traditional logistic regression, evidenced by improved accuracy and area under the curve (AUC). This finding is consistent with recent research demonstrating the potential of machine learning in enhancing risk prediction accuracy, particularly in handling complex, nonlinear relationships between variables [28,29].

Our study extends the body of work on lung cancer risk prediction by demonstrating that machine learning models might achieve greater performance than traditional statistical methods. Previous lung cancer prediction models, such as the Liverpool Lung Project (LLP) and the Prostate, Lung, Colorectal, and Ovarian (PLCO) Cancer Screening Trial models, have traditionally relied on logistic regression approaches. The LLP model, for instance, includes factors like age, smoking history, family history of lung cancer, and asbestos exposure [30]. In addition, current lung cancer risk prediction models, including LLP, PLCO, LCDRAT, LCRAT, Bach, HUNT, OWL, UCLD, and UCLI, have demonstrated similar discriminatory power in most countries. LCDRAT and UCLD, unlike others, predict the risk of mortality rather than the risk of incidence. Most models identify more future lung cancer cases than the current classification criteria [31]. The accuracy of the models generally exceeds 0.7, with representative models such as the LLP and PLCO models scoring 0.70, the Bach model at 0.72, the LCDRAT model at 0.78, and the LCRAT model at 0.80 (PLCO) [32]. While these models have shown moderate accuracy with AUCs around 0.70–0.75 [30,33], they may not capture complex relationships as effectively as modern machine learning techniques. Our work leveraged a wide range of demographic, clinical, and behavioral variables collected through standardized epidemiological questionnaires. These variables go beyond what is typically included in classical models like LLP and PLCO, potentially capturing subtler risk signals and improving predictive performance.

In comparison, the machine learning models used in our study, particularly the stacking model, achieved higher AUC values, highlighting their capacity to manage larger datasets and model nonlinear interactions more effectively. The PLCO model, which incorporates additional variables like COPD history, has shown AUCs between 0.68 and 0.73 [31,34]. Despite their reliability, both LLP and PLCO models are limited by their relatively rigid structure, as they cannot adjust dynamically to new data, which is a significant advantage offered by machine learning models. This might imply that stacking ensemble models significantly achieved good performance, which suggests that ML models may have strong potential for enhancing early lung cancer risk assessment.

Recent efforts to improve lung cancer prediction, such as OWL (Optimized early Warning model for Lung cancer) [11], have demonstrated the superiority of machine learning approaches like XGBoost over traditional models. For example, OWL achieved an AUC of 0.85, surpassing both the PLCO and LLP models in independent validations [11]. This further supports the findings of our study, which also demonstrate that machine learning models provide good performance and calibration. Moreover, while many machine learning models, including OWL, function as “black boxes” with limited interpretability. This interpretability is crucial in clinical settings, where understanding the contribution of individual risk factors is necessary for informed decision-making [33].

Early detection is crucial, as lung cancer is often diagnosed at advanced stages, limiting treatment options and resulting in poor survival outcomes. Our study offers important implications for lung cancer risk prediction, particularly in improving the early identification of high-risk individuals. A key strength of this study lies in its large-scale sample size and the comprehensiveness of the dataset, which integrates diverse demographic, behavioral, and clinical features. Firstly, our findings highlight notable regional variations in risk factor distributions and model performance, underscoring the need for population-specific tools in Chinese lung cancer screening programs. Although the differences in AUC values between models appear modest, statistical analysis using the DeLong test confirms that our stacking model achieves a significantly improved performance compared to certain baseline models. Nonetheless, we acknowledge that model evaluation should not rely solely on AUC. A more comprehensive assessment should incorporate additional performance metrics, including the F1-score, precision, recall, and calibration curves, to ensure both predictive accuracy and clinical utility. Secondly, the superior performance of machine learning models, such as LightGBM and stacking, suggests that their integration into clinical practice could significantly enhance the precision of lung cancer screening programs. Recent studies have demonstrated that AI-driven models can effectively support risk stratification, enabling more personalized screening strategies. These approaches allow for the targeted identification of high-risk individuals while minimizing unnecessary procedures for those at lower risk [35,36]. In addition, machine learning algorithms have shown considerable potential in improving the accuracy of lung nodule detection and characterization during screening, particularly when combined with low-dose computed tomography (LDCT) scans. By leveraging large-scale datasets, AI could facilitate the development of tailored screening protocols based on individual patient risk profiles, ultimately increasing early detection rates [35]. Moreover, this study used stacking models integrating multiple base learners into a more robust meta-model, thereby improving overall predictive performance. Thirdly, stacking enhances the generalization capacity of the model, making it more effective in addressing a wide range of predictive tasks [37,38,39].

However, several limitations must be acknowledged. First, although machine learning models demonstrate superior predictive accuracy, their interpretability remains a significant challenge, particularly in clinical settings. Explainable AI (XAI) techniques have emerged as vital tools in addressing this issue, as they help clinicians understand and interpret the predictions made by complex models. This transparency is essential for fostering trust and facilitating the adoption of high-performing machine learning models in real-world clinical practice [35,36]. By bridging the gap between model performance and clinical applicability, XAI not only enhances clinician confidence but also supports improved patient outcomes through more personalized and timely interventions. Nonetheless, we recognize that interpretability remains a concern, especially for stacked models. These ensemble models integrate multiple base learners and often operate as “black boxes,” making it difficult to discern the specific contribution of each individual model within the ensemble. In contrast, traditional statistical approaches, such as logistic regression, provide more straightforward interpretations of predictor variables, an aspect that remains crucial for clinical decision-making [28]. Second, our study was based on a single dataset, and external validation using independent cohorts from different regions or populations is necessary to assess the generalizability and robustness of the proposed models. Finally, potential biases stemming from self-reported questionnaire data and the imputation methods used for handling missing values might affect the overall reliability of results [28]. These limitations underscore the need for cautious interpretation of our findings.

Future research should focus on validating these models in external datasets to assess their generalizability across different populations and healthcare settings. Additionally, integrating other data sources, such as genetic factors, circulating biomarkers, or environmental factors, could further improve the predictive power of these models. Moreover, incorporating techniques for handling censored data, as seen in survival models, may enhance the applicability of machine learning models to time-to-event outcomes, which is particularly relevant for diseases like cancer [28,29].

## 5. Conclusions

This study demonstrates that machine learning models, particularly stacking models and lightGBM, significantly enhance lung cancer risk prediction compared to traditional methods. These models outperformed logistic regression in terms of accuracy and AUC, showing their ability to capture complex relationships in health data. However, improving model interpretability remains essential for clinical adoption. Future efforts should focus on explainable AI techniques and external validation across diverse populations. With continued progress, machine learning could transform lung cancer screening, enabling earlier interventions and better patient outcomes.

## Figures and Tables

**Table 1:** Baseline characteristics of the study participants.

Characteristics	Case (*N* = 5421)	Control (*N* = 10,831)	*p*-Value
Age, median [IQR], years	58.1 [48.3, 66.2]	58.1 [48.4, 66.4]	0.752
Sex, No. (%)			0.949
Male	1986 (36.6%)	3975 (36.7%)	
Female	3435 (63.4%)	6856 (63.3%)	
Smoking status, No. (%)			0.994
Current	420 (7.7%)	838 (7.7%)	
Former	925 (17.1%)	1841 (17.0%)	
Never	4076 (75.2%)	8152 (75.3%)	
Education, No. (%)			<0.001
Uneducated	465 (8.6%)	2218 (20.5%)	
Primary school	1309 (24.2%)	2891 (26.7%)	
Middle school	1510 (27.9%)	2926 (27.0%)	
High school	873 (16.1%)	1073 (9.9%)	
Technical school	567 (10.5%)	438 (4.0%)	
College	595 (11.0%)	866 (8.0%)	
Graduate school	88 (1.6%)	379 (3.5%)	
Other	6 (0.1%)	36 (0.3%)	
Income, No. (%)			<0.001
<50,000	898 (17.8%)	2374 (29.8%)	
50,000–100,000	1457 (28.9%)	2947 (37.0%)	
110,000–200,000	1280 (25.4%)	1514 (19.0%)	
210,000–300,000	672 (13.3%)	399 (5.0%)	
>300,000	730 (14.5%)	730 (9.2%)	
Second-hand smoke, No. (%)			<0.001
No	2891 (55.3%)	7629 (71.9%)	
Yes	2337 (44.7%)	2985 (28.1%)	
Live with smokers, No. (%)			<0.001
Never	3183 (60.0%)	8341 (77.9%)	
Former	841 (15.9%)	993 (9.3%)	
Current	1279 (24.1%)	1372 (12.8%)	
Taste, No. (%)			<0.001
Heavy	1131 (21.0%)	1692 (15.7%)	
Moderate	2645 (49.2%)	6367 (59.1%)	
Light	1600 (29.8%)	2720 (25.2%)	
Spiciness, No. (%)			<0.001
Hot	69 (1.3%)	150 (1.4%)	
Medium	393 (7.3%)	1185 (11.1%)	
Mild	2025 (37.7%)	5532 (51.7%)	
Not spicy	2879 (53.7%)	3829 (35.8%)	
Leftovers, No. (%)			<0.001
Everyday	595 (11.1%)	706 (7.1%)	
3–5 times per week	1099 (20.5%)	1260 (12.6%)	
1–2 times per week	1395 (26.1%)	3412 (34.2%)	
Seldom	2259 (42.2%)	4603 (46.1%)	
Dietary preference, No. (%)			<0.001
Meat	631 (12.1%)	1133 (10.6%)	
Balance	3725 (71.2%)	8295 (77.4%)	
Vegetarian	879 (16.8%)	1294 (12.1%)	
Difficult to fall asleep, No. (%)			<0.001
Never	3558 (75.5%)	8027 (75.2%)	
<1 times per week	543 (11.5%)	1252 (11.7%)	
1–2 times per week	541 (11.5%)	944 (8.8%)	
≥3 times per week	70 (1.5%)	453 (4.2%)	
Difficult to breathe, No. (%)			<0.001
Never	4471 (91.5%)	9875 (92.5%)	
<1 times per week	192 (3.9%)	448 (4.2%)	
1–2 times per week	190 (3.9%)	268 (2.5%)	
≥3 times per week	33 (0.7%)	84 (0.8%)	
Cough, No. (%)			<0.001
Never	2491 (67.9%)	7397 (72.6%)	
<1 times per week	441 (12.0%)	957 (9.4%)	
1–2 times per week	613 (16.7%)	1088 (10.7%)	
≥3 times per week	123 (3.4%)	747 (7.3%)	
Pain, No. (%)			<0.001
Never	4712 (93.8%)	9257 (87.0%)	
<1 times per week	163 (3.2%)	640 (6.0%)	
1–2 times per week	127 (2.5%)	501 (4.7%)	
≥3 times per week	19 (0.4%)	245 (2.3%)	
Exercise frequency, No. (%)			<0.001
Never	2543 (47.6%)	7628 (70.8%)	
1–3 times per month	320 (6.0%)	514 (4.8%)	
1–2 times per week	458 (8.6%)	752 (7.0%)	
3–5 times per week	366 (6.9%)	501 (4.7%)	
Everyday	1650 (30.9%)	1375 (12.8%)	
Chronic bronchitis, No. (%)			0.05
No	5370 (99.1%)	10,761 (99.4%)	
Yes	51 (0.9%)	70 (0.6%)	
Asthma, No. (%)			<0.001
No	5385 (99.3%)	10,803 (99.7%)	
Yes	36 (0.7%)	28 (0.3%)	
Chronic obstructive pulmonary disease, No. (%)			0.003
No	5412 (99.8%)	10,779 (99.5%)	
Yes	9 (0.2%)	52 (0.5%)	
Tuberculosis, No. (%)			<0.001
No	5361 (98.9%)	10,790 (99.6%)	
Yes	60 (1.1%)	41 (0.4%)	
Pulmonary nodule, No. (%)			<0.001
No	4433 (81.8%)	10,765 (99.4%)	
Yes	988 (18.2%)	66 (0.6%)	
Family history *, No. (%)			<0.001
No	4163 (76.8%)	10,368 (95.7%)	
Yes	1258 (23.2%)	463 (4.3%)	
Heavy metal, No. (%)			<0.001
No	5335 (98.4%)	10,762 (99.4%)	
Yes	86 (1.6%)	69 (0.6%)	
Diesel, No. (%)			<0.001
No	5360 (98.9%)	10,794 (99.7%)	
Yes	61 (1.1%)	37 (0.3%)	
Coal dust, No. (%)			<0.001
No	5360 (98.9%)	10,796 (99.7%)	
Yes	61 (1.1%)	35 (0.3%)	
Asbestos, No. (%)			0.662
No	5405 (99.7%)	10,793 (99.6%)	
Yes	16 (0.3%)	38 (0.4%)	
Physical activity, No. (%)			<0.001
Inactive	2810 (52.3%)	7873 (75.1%)	
Low active	501 (9.3%)	545 (5.2%)	
Moderate active	1311 (24.4%)	1270 (12.1%)	
High active	747 (13.9%)	802 (7.6%)	
Drinking status, No. (%)			<0.001
Never	3928 (72.7%)	7959 (73.6%)	
Current	1091 (20.2%)	2390 (22.1%)	
Former	385 (7.1%)	461 (4.3%)	
Height, median [IQR], cm	161.0 [156.5, 168.0]	160.0 [155.0, 166.0]	<0.001
Weight, median [IQR], kg	60.0 [53.0, 67.3]	60.0 [53.0, 66.4]	0.002
Weight change ^†^, median [IQR]	0.00 [−0.5, 0.5]	0.0 [0.0, 2.0]	<0.001
Pack year ^‡^, median [IQR], years	30.0 [15.0, 46.0]	29.3 [12.5, 45.0]	0.03

* Either parents, siblings, or children were diagnosed with malignant tumor before baseline survey. ^†^ The difference in weight at baseline and three years ago. ^‡^ The median and IQR are calculated for current and former smokers.

**Table 2:** Performance of machine learning-based models in training, validation and test datasets.

Model	All	Never	Current	Former
LLP *				
Training	0.636 (0.627–0.645)	0.635 (0.627–0.643)	0.656 (0.620–0.692)	0.634 (0.610–0.659)
Validation	0.631 (0.605–0.657)	0.623 (0.601–0.645)	0.604 (0.494–0.713)	0.639 (0.563–0.716)
Test	0.647 (0.620–0.673)	0.636 (0.613–0.659)	0.635 (0.508–0.762)	0.653 (0.585–0.721)
PLCO ^†^				
Training	0.678 (0.668–0.687)	0.692 (0.681–0.703)	0.690 (0.656–0.725)	0.687 (0.664–0.710)
Validation	0.661 (0.633–0.690)	0.677 (0.645–0.709)	0.603 (0.492–0.713)	0.673 (0.597–0.748)
Test	0.662 (0.633–0.691)	0.676 (0.643–0.709)	0.606 (0.486–0.726)	0.683 (0.614–0.752)
LightGBM				
Training	0.922 (0.918–0.927)	0.935 (0.930–0.939)	0.995 (0.992–0.998)	0.873 (0.858–0.889)
Validation	0.880 (0.863–0.897)	0.877 (0.858–0.897)	0.864 (0.802–0.926)	0.856 (0.808–0.903)
Test	0.884 (0.867–0.901)	0.897 (0.879–0.915)	0.800 (0.719–0.880)	0.809 (0.757–0.861)
MLP				
Training	0.904 (0.898–0.909)	0.894 (0.888–0.900)	0.858 (0.831–0.885)	0.894 (0.881–0.908)
Validation	0.864 (0.845–0.883)	0.872 (0.852–0.893)	0.889 (0.826–0.952)	0.817 (0.764–0.869)
Test	0.877 (0.860–0.894)	0.893 (0.875–0.912)	0.822 (0.749–0.895)	0.799 (0.745–0.852)
Stacking *				
Training	0.915 (0.910–0.920)	0.920 (0.915–0.926)	0.900 (0.879–0.921)	0.928 (0.917–0.939)
Validation	0.881 (0.864–0.899)	0.881 (0.862–0.900)	0.900 (0.842–0.959)	0.850 (0.801–0.898)
Test	0.887 (0.870–0.903)	0.901 (0.883–0.918)	0.837 (0.769–0.906)	0.814 (0.763–0.864)

* LLP = the Liverpool Lung Project; variables in the LLP model include smoking duration, asbestos, family history of lung cancer, prior diagnosis of malignant tumor and prior diagnosis of any lung disease including chronic bronchitis, COPD, asthma, pulmonary nodule and tuberculosis. ^†^ PLCO = the Prostate, Lung, Colorectal and Ovarian cancer screening trial; variables in the PLCO model include age, education, BMI, family lung disease history, COPD, packyear and smoking years and smoking status. The stacking model was constructed with five base models and a logistic regression meta-learner: for full data and never-smokers, the five base models include MLP, LightGBM, GBDT, SVM and XGBoost; for current smokers, the five base models include RF, GBDT, SVM, LogiR and XGBoost; for former smokers, the five base models include RF, MLP, SVM, lightGBM and XGBoost. AUC: area under the curve; LogiR: regularized logistic regression; RF: random forest; LightGBM: light gradient boosting machine; ET: extra trees; XGBoost: extreme gradient boosting; AdaBoost: adaptive boosting; GBDT: gradient boosting decision tree; SVM: support vector machine; MLP: multilayer perceptron.

**Table 3:** Multivariate logistic model with ORs for lung cancer by smoking status.

	All	Never	Current	Former
Risk Factor	OR (95% CI)	OR (95% CI)	OR (95% CI)	OR (95% CI)
Education, No. (%)				
Uneducated	1.00 (reference)	1.00 (reference)	1.00 (reference)	1.00 (reference)
Primary school	1.94 (1.68–2.26)	1.98 (1.67–2.35)	2.94 (1.45–5.99)	1.52 (1.02–2.26)
Middle school	2.32 (1.98–2.71)	2.47 (2.06–2.96)	3.25 (1.58–6.69)	1.50 (0.99–2.26)
High school	3.25 (2.71–3.90)	3.87 (3.13–4.79)	4.59 (2.08–10.14)	1.49 (0.93–2.38)
Technical school	4.63 (3.70–5.80)	6.45 (4.95–8.40)	8.14 (2.94–22.54)	1.08 (0.62–1.89)
College	2.12 (1.68–2.67)	2.85 (2.18–3.72)	2.41 (0.83–6.95)	0.35 (0.18–0.68)
Graduate school	0.60 (0.42–0.86)	0.72 (0.48–1.07)	1.63 (0.22–12.33)	0.14 (0.04–0.47)
Other	1.13 (0.43–2.92)	1.13 (0.39–3.28)	NA	NA
*p* for interaction	<0.001			
Income, No. (%)				
<50,000	1.00 (reference)	1.00 (reference)	1.00 (reference)	1.00 (reference)
50,000–100,000	1.38 (1.23–1.55)	1.29 (1.12–1.48)	1.56 (1.01–2.42)	1.65 (1.26–2.16)
110,000–200,000	2.17 (1.89–2.48)	2.11 (1.80–2.48)	2.04 (1.22–3.40)	2.48 (1.80–3.41)
210,000–300,000	4.83 (4.02–5.80)	4.51 (3.64–5.60)	5.60 (2.65–11.85)	6.40 (4.06–10.11)
>300,000	2.83 (2.37–3.39)	2.61 (2.11–3.23)	3.60 (1.70–7.62)	3.85 (2.54–5.84)
*p* for interaction	0.256			
Second-hand smoke, No. (%)				
No	1.00 (reference)	1.00 (reference)	1.00 (reference)	1.00 (reference)
Yes	1.30 (1.18–1.44)	1.50 (1.32–1.69)	1.26 (0.90–1.78)	0.89 (0.71–1.10)
*p* for interaction	<0.001			
Live with smokers, No. (%)				
Never	1.00 (reference)	1.00 (reference)	1.00 (reference)	1.00 (reference)
Former	1.55 (1.35–1.78)	1.97 (1.67–2.33)	0.73 (0.39–1.36)	0.70 (0.50–0.97)
Current	2.06 (1.82–2.34)	2.04 (1.77–2.36)	2.57 (1.50–4.41)	2.10 (1.39–3.17)
*p* for interaction	<0.001			
Taste, No. (%)				
Heavy	1.00 (reference)	1.00 (reference)	1.00 (reference)	1.00 (reference)
Moderate	0.57 (0.50–0.63)	0.61 (0.53–0.70)	0.57 (0.40–0.82)	0.46 (0.36–0.59)
Light	0.68 (0.59–0.77)	0.71 (0.60–0.83)	0.54 (0.33–0.88)	0.60 (0.45–0.80)
*p* for interaction	0.042			
Spiciness, No. (%)				
Hot	1.00 (reference)	1.00 (reference)	1.00 (reference)	1.00 (reference)
Medium	0.67 (0.46–0.99)	0.57 (0.35–0.93)	0.59 (0.20–1.72)	0.94 (0.43–2.02)
Mild	0.84 (0.59–1.21)	0.71 (0.44–1.13)	0.77 (0.28–2.08)	1.11 (0.53–2.30)
Not spicy	2.13 (1.49–3.06)	1.93 (1.21–3.08)	1.72 (0.62–4.74)	2.23 (1.07–4.65)
*p* for interaction	0.25			
Leftovers, No. (%)				
Everyday	1.00 (reference)	1.00 (reference)	1.00 (reference)	1.00 (reference)
3–5 times per week	1.00 (0.84–1.19)	1.06 (0.86–1.31)	1.18 (0.58–2.38)	0.86 (0.59–1.25)
1–2 times per week	0.43 (0.36–0.50)	0.41 (0.34–0.51)	0.55 (0.28–1.08)	0.47 (0.33–0.67)
Seldom	0.40 (0.34–0.47)	0.43 (0.35–0.52)	0.44 (0.23–0.85)	0.33 (0.24–0.47)
*p* for interaction	0.086			
Dietary preference, No. (%)				
Meat	1.00 (reference)	1.00 (reference)	1.00 (reference)	1.00 (reference)
Balance	1.05 (0.91–1.20)	1.12 (0.93–1.35)	0.87 (0.57–1.34)	0.98 (0.75–1.27)
Vegetarian	1.27 (1.07–1.51)	1.42 (1.14–1.76)	0.73 (0.36–1.48)	1.02 (0.70–1.50)
*p* for interaction	0.079			
Difficult to fall asleep, No. (%)				
Never	1.00 (reference)	1.00 (reference)	1.00 (reference)	1.00 (reference)
<1 times per week	0.87 (0.76–1.00)	0.90 (0.77–1.06)	0.72 (0.40–1.29)	0.84 (0.60–1.18)
1–2 times per week	1.06 (0.92–1.23)	1.17 (0.99–1.38)	0.46 (0.23–0.94)	0.73 (0.49–1.10)
≥3 times per week	0.21 (0.15–0.29)	0.22 (0.15–0.32)	0.49 (0.14–1.67)	0.07 (0.02–0.22)
*p* for interaction	<0.001			
Difficult to breathe, No. (%)				
Never	1.00 (reference)	1.00 (reference)	1.00 (reference)	1.00 (reference)
<1 times per week	0.96 (0.76–1.23)	0.88 (0.66–1.17)	1.90 (0.81–4.45)	1.05 (0.60–1.81)
1–2 times per week	1.99 (1.54–2.57)	2.32 (1.70–3.16)	1.39 (0.51–3.84)	1.36 (0.76–2.45)
≥3 times per week	0.95 (0.52–1.75)	1.51 (0.71–3.24)	0.25 (0.02–2.90)	0.73 (0.22–2.43)
*p* for interaction	0.048			
Cough, No. (%)				
Never	1.00 (reference)	1.00 (reference)	1.00 (reference)	1.00 (reference)
<1 times per week	1.11 (0.95–1.29)	1.16 (0.97–1.39)	0.93 (0.50–1.72)	0.98 (0.70–1.37)
1–2 times per week	1.15 (1.01–1.32)	1.26 (1.07–1.48)	0.88 (0.55–1.40)	0.97 (0.73–1.29)
≥3 times per week	0.39 (0.32–0.49)	0.26 (0.19–0.37)	0.45 (0.25–0.82)	0.45 (0.32–0.63)
*p* for interaction	0.002			
Pain, No. (%)				
Never	1.00 (reference)	1.00 (reference)	1.00 (reference)	1.00 (reference)
<1 times per week	0.46 (0.37–0.58)	0.47 (0.36–0.62)	0.79 (0.34–1.84)	0.30 (0.17–0.53)
1–2 times per week	0.39 (0.31–0.51)	0.40 (0.30–0.54)	0.63 (0.23–1.72)	0.31 (0.15–0.60)
≥3 times per week	0.15 (0.08–0.26)	0.17 (0.09–0.33)	0.14 (0.02–1.32)	0.04 (0.01–0.25)
*p* for interaction	0.087			
Exercise frequency, No. (%)				
Never	1.00 (reference)	1.00 (reference)	1.00 (reference)	1.00 (reference)
1–3 times per month	2.09 (1.65–2.65)	2.08 (1.59–2.72)	3.48 (1.09–11.08)	1.80 (0.96–3.39)
1–2 times per week	2.31 (1.75–3.05)	2.29 (1.67–3.15)	6.48 (1.91–21.92)	1.93 (0.88–4.22)
3–5 times per week	2.89 (2.13–3.93)	3.06 (2.15–4.36)	5.43 (1.39–21.21)	2.15 (0.95–4.89)
Everyday	5.76 (4.35–7.63)	5.78 (4.18–7.98)	24.21 (6.50–90.19)	5.07 (2.41–10.67)
*p* for interaction	0.001			
Chronic bronchitis, No. (%)				
No	1.00 (reference)	1.00 (reference)	1.00 (reference)	1.00 (reference)
Yes	0.91 (0.56–1.48)	0.99 (0.40–2.41)	12.45 (0.84–184.65)	0.97 (0.52–1.81)
*p* for interaction	0.164			
Asthma, No. (%)				
No	1.00 (reference)	1.00 (reference)	1.00 (reference)	1.00 (reference)
Yes	2.52 (1.34–4.74)	4.27 (1.84–9.92)	0.78 (0.11–5.29)	1.37 (0.40–4.68)
*p* for interaction	0.113			
Chronic obstructive pulmonary disease, No. (%)				
No	1.00 (reference)	1.00 (reference)	1.00 (reference)	1.00 (reference)
Yes	0.23 (0.09–0.59)	0.32 (0.06–1.63)	1.88 (0.19–18.51)	0.16 (0.03–0.70)
*p* for interaction	0.168			
Tuberculosis, No. (%)				
No	1.00 (reference)	1.00 (reference)	1.00 (reference)	1.00 (reference)
Yes	3.69 (2.23–6.12)	5.01 (2.59–9.69)	26.04 (1.55–437.05)	1.47 (0.55–3.90)
*p* for interaction	0.018			
Pulmonary nodule, No. (%)				
No	1.00 (reference)	1.00 (reference)	1.00 (reference)	1.00 (reference)
Yes	41.32 (31.40–54.36)	42.82 (30.86–59.39)	98.10 (26.91–357.66)	33.75 (18.67–61.00)
*p* for interaction	0.179			
Family history *, No. (%)				
No	1.00 (reference)	1.00 (reference)	1.00 (reference)	1.00 (reference)
Yes	5.04 (4.41–5.76)	5.86 (4.96–6.93)	6.29 (3.75–10.55)	3.22 (2.44–4.23)
*p* for interaction	<0.001			
Heavy metal, No. (%)				
No	1.00 (reference)	1.00 (reference)	1.00 (reference)	1.00 (reference)
Yes	1.97 (1.31–2.96)	1.60 (0.92–2.77)	2.13 (0.54–8.50)	3.05 (1.49–6.27)
*p* for interaction	0.378			
Diesel, No. (%)				
No	1.00 (reference)	1.00 (reference)	1.00 (reference)	1.00 (reference)
Yes	2.20 (1.29–3.73)	2.46 (1.18–5.14)	3.23 (0.62–16.90)	2.37 (0.98–5.74)
*p* for interaction	0.735			
Coal dust, No. (%)				
No	1.00 (reference)	1.00 (reference)	1.00 (reference)	1.00 (reference)
Yes	2.73 (1.61–4.65)	4.00 (1.88–8.53)	15.70 (1.81–135.82)	1.18 (0.46–3.03)
*p* for interaction	0.013			
Asbestos, No. (%)				
No	1.00 (reference)	1.00 (reference)	1.00 (reference)	1.00 (reference)
Yes	0.97 (0.46–2.03)	1.02 (0.43–2.44)	3.08 (0.16–58.33)	0.65 (0.11–3.82)
*p* for interaction	0.561			
Physical activity, No. (%)				
Inactive	1.00 (reference)	1.00 (reference)	1.00 (reference)	1.00 (reference)
Low active	0.58 (0.44–0.77)	0.61 (0.44–0.83)	0.28 (0.08–0.96)	0.54 (0.25–1.18)
Moderate active	0.47 (0.36–0.62)	0.52 (0.38–0.71)	0.11 (0.03–0.39)	0.44 (0.21–0.91)
High active	0.39 (0.29–0.52)	0.42 (0.30–0.58)	0.08 (0.02–0.33)	0.40 (0.19–0.85)
*p* for interaction	<0.001			
Drinking status, No. (%)				
Never	1.00 (reference)	1.00 (reference)	1.00 (reference)	1.00 (reference)
Current	0.93 (0.83–1.04)	0.76 (0.65–0.89)	1.68 (1.21–2.35)	0.99 (0.79–1.25)
Former	1.63 (1.35–1.97)	1.88 (1.36–2.62)	3.63 (1.80–7.31)	1.39 (1.06–1.82)
*p* for interaction	<0.001			
Height, cm	1.02 (1.01–1.03)	1.02 (1.01–1.03)	1.03 (1.00–1.06)	1.05 (1.02–1.07)
*p* for interaction	0.079			
Weight, kg	1.00 (1.00–1.01)	1.00 (1.00–1.01)	1.01 (0.99–1.03)	1.00 (0.99–1.01)
*p* for interaction	0.081			
Weight change ^†^, kg	0.94 (0.93–0.96)	0.93 (0.92–0.95)	0.93 (0.87–0.99)	0.97 (0.94–1.00)
*p* for interaction	0.043			
Pack year ^‡^, years	1.01 (1.00–1.01)	NA	1.00 (1.00–1.01)	1.00 (1.00–1.01)
*p* for interaction	0.625			

* Either parents, siblings, or children were diagnosed with malignant tumor before baseline survey. ^†^ The difference in weight at baseline and three years ago. ^‡^ The median and IQR were calculated for current and former smokers. NA: Not applicable. The variable is not included in the model. Note: *p* value for interaction was used to describe the comparison between smoking subgroups (never-smokers, current smokers and former smokers).

## Data Availability

The data supporting the findings of this study are available from X.W., but restrictions apply to their availability. These data were used under license for the current study and are not publicly accessible. However, data and code can be made available by the authors upon reasonable request and with permission from X.W.

## Supplementary Materials

The following supporting information can be downloaded at: https://www.mdpi.com/article/10.3390/cancers17101651/s1, Figure S1: Calibration curves of base and stacking machine learning models for predictions of lung cancer in test dataset. A. CALIBRATION curves of MLP, LightGBM, GBDT, SVM and XGBoost and Stacking model in full data. B. CALIBRATION curves of MLP, LightGBM, GBDT, SVM and XGBoost and Stacking model in never smokers. C. CALIBRATION curves of AdaBoost, GBDT, SVM, MLP and XGBoost and Stacking model in current smokers. D. CALIBRATION curves of LogiR, GBDT, RF, lightGBM and XGBoost and Stacking model in former smokers. Table S1: Performance of machine learning-based models for all data. Table S2: Performance of machine learning-based models for never smokers. Table S3: Performance of machine learning-based models for current smokers. Table S4: Performance of machine learning-based models for former smokers.
