# Supplementary material for: Improving Lung Cancer Risk Prediction Using Machine Learning: A Comparative Analysis of Stacking Models and Traditional Approaches
Source: Cancers (Basel). 2025 May 13;17(10):1651. doi: 10.3390/cancers17101651 (PMC12109916; doi:10.3390/cancers17101651)
Supplement: Supplementary file 1 [file cancers-17-01651-s001.zip › cancers-3577085-supplementary.pdf]

Table S1. Performance of machine learning-based models for all data

	Recall	Specificity	AUC (95%CI)	Brier score	F1	Accuracy
**LogiR**						
Training	0.752	0.805	0.857 (0.850 - 0.864)	0.151	0.702	0.788
Validation	0.740	0.813	0.858 (0.839 - 0.878)	0.150	0.702	0.788
Test	0.758	0.812	0.858 (0.839 - 0.878)	0.151	0.71	0.794
**RF**						
Training	0.786	0.835	0.895 (0.889 - 0.901)	0.148	0.743	0.818
Validation	0.748	0.807	0.858 (0.839 - 0.878)	0.163	0.703	0.787
Test	0.745	0.791	0.865 (0.846 - 0.883)	0.161	0.689	0.776
**LightGBM**						
Training	0.829	0.85	0.922 (0.918 - 0.927)	0.116	0.779	0.843
Validation	0.753	0.823	0.880 (0.863 - 0.897)	0.137	0.717	0.799
Test	0.771	0.815	0.884 (0.867 - 0.901)	0.137	0.721	0.801
**ET**						
Training	0.758	0.781	0.851 (0.844 - 0.858)	0.175	0.69	0.773
Validation	0.753	0.778	0.839 (0.819 - 0.859)	0.180	0.688	0.770
Test	0.751	0.758	0.838 (0.818 - 0.859)	0.180	0.672	0.756
**XGBoost**						
Training	0.615	0.925	0.891 (0.885 - 0.897)	0.125	0.697	0.821
Validation	0.590	0.921	0.870 (0.851 - 0.888)	0.136	0.676	0.81
Test	0.613	0.909	0.876 (0.859 - 0.893)	0.133	0.682	0.81
**AdaBoost**						
Training	0.477	0.942	0.852 (0.845 - 0.859)	0.238	0.599	0.787
Validation	0.468	0.942	0.854 (0.834 - 0.874)	0.238	0.591	0.782
Test	0.474	0.937	0.851 (0.831 - 0.871)	0.238	0.593	0.783
**GBDT**						
Training	0.62	0.922	0.888 (0.883 - 0.894)	0.125	0.699	0.822
Validation	0.596	0.917	0.871 (0.853 - 0.889)	0.134	0.678	0.809
Test	0.616	0.908	0.872 (0.854 - 0.890)	0.134	0.684	0.810
**SVM**						
Training	0.836	0.842	0.915 (0.910 - 0.920)	0.108	0.777	0.840
Validation	0.770	0.811	0.874 (0.856 - 0.892)	0.133	0.718	0.797
Test	0.773	0.812	0.876 (0.858 - 0.893)	0.133	0.719	0.799
**MLP**						
Training	0.687	0.911	0.904 (0.898 - 0.909)	0.115	0.737	0.836
Validation	0.629	0.887	0.864 (0.845 - 0.883)	0.139	0.679	0.800
Test	0.672	0.882	0.877 (0.860 - 0.894)	0.132	0.704	0.812
**Stacking***						
Training	0.792	0.869	0.915 (0.910 - 0.920)	0.149	0.771	0.843
Validation	0.742	0.841	0.881 (0.864 - 0.899)	0.160	0.722	0.808
Test	0.755	0.840	0.887 (0.870 - 0.903)	0.159	0.728	0.812

The Stacking model was constructed by five base models (MLP, LightGBM, GBDT, SVM and XGBoost) and a logistic regression meta-learner. AUC: area under the curve; LogiR: logistic regression; RF: random forest; LightGBM: light gradient boosting machine; ET: extra trees; XGBoost: extreme gradient boosting; AdaBoost: adaptive boosting; GBDT: gradient boosting decision tree; SVM: Support vector machine; MLP: multilayer perceptron

Table S2. Performance of machine learning-based models for never smokers

	Recall	Specificity	AUC (95%CI)	Brier score	F1	Accuracy
**LogiR**						
Training	0.763	0.812	0.870 (0.862 - 0.877)	0.144	0.714	0.796
Validation	0.751	0.797	0.858 (0.836 - 0.880)	0.152	0.699	0.782
Test	0.788	0.816	0.876 (0.855 - 0.897)	0.141	0.726	0.807
**RF**						
Training	0.800	0.847	0.906 (0.900 - 0.913)	0.139	0.76	0.831
Validation	0.763	0.793	0.858 (0.837 - 0.879)	0.160	0.704	0.783
Test	0.793	0.798	0.881 (0.861 - 0.900)	0.152	0.717	0.796
**LightGBM**						
Training	0.853	0.855	0.935 (0.930 - 0.939)	0.107	0.797	0.854
Validation	0.759	0.809	0.877 (0.858 - 0.897)	0.141	0.712	0.792
Test	0.801	0.815	0.897 (0.879 - 0.915)	0.130	0.733	0.810
**ET**						
Training	0.771	0.788	0.868 (0.860 - 0.875)	0.169	0.702	0.782
Validation	0.751	0.771	0.841 (0.819 - 0.863)	0.179	0.683	0.764
Test	0.765	0.759	0.854 (0.833 - 0.876)	0.175	0.675	0.761
**XGBoost**						
Training	0.642	0.930	0.902 (0.896 - 0.908)	0.118	0.721	0.834
Validation	0.595	0.907	0.872 (0.852 - 0.892)	0.136	0.669	0.801
Test	0.643	0.911	0.888 (0.869 - 0.907)	0.125	0.703	0.824
**AdaBoost**						
Training	0.515	0.941	0.864 (0.857 - 0.872)	0.237	0.631	0.799
Validation	0.502	0.929	0.857 (0.836 - 0.879)	0.237	0.612	0.785
Test	0.503	0.938	0.871 (0.850 - 0.892)	0.236	0.616	0.796
**GBDT**						
Training	0.647	0.929	0.904 (0.898 - 0.910)	0.116	0.724	0.835
Validation	0.627	0.909	0.876 (0.856 - 0.895)	0.134	0.695	0.814
Test	0.645	0.920	0.895 (0.877 - 0.913)	0.119	0.713	0.831
**SVM**						
Training	0.849	0.842	0.921 (0.915 - 0.926)	0.105	0.785	0.844
Validation	0.783	0.806	0.873 (0.853 - 0.894)	0.136	0.724	0.798
Test	0.783	0.804	0.889 (0.871 - 0.908)	0.124	0.715	0.797
**MLP**						
Training	0.66	0.909	0.894 (0.888 - 0.900)	0.12	0.717	0.826
Validation	0.646	0.889	0.872 (0.852 - 0.893)	0.134	0.694	0.807
Test	0.653	0.900	0.893 (0.875 - 0.912)	0.121	0.701	0.820
**Stacking***						
Training	0.792	0.869	0.920 (0.915 - 0.926)	0.152	0.772	0.843
Validation	0.754	0.834	0.881 (0.862 - 0.900)	0.165	0.725	0.807
Test	0.773	0.843	0.901 (0.883 - 0.918)	0.159	0.736	0.820

The Stacking model was constructed by five base models (MLP, LightGBM, GBDT, SVM and XGBoost) and a logistic regression meta-learner. AUC: area under the curve; LogiR: logistic regression; RF: random forest; LightGBM: light

gradient boosting machine; ET: extra trees; XGBoost: extreme gradient boosting; AdaBoost: adaptive boosting; GBDT: gradient boosting decision tree; SVM: Support vector machine; MLP: multilayer perceptron

Table S3. Performance of machine learning-based models for current smokers

	Recall	Specificity	AUC (95%CI)	Brier score	F1	Accuracy
**LogiR**						
Training	0.764	0.816	0.859 (0.833 - 0.885)	0.152	0.714	0.799
Validation	0.825	0.828	0.892 (0.825 - 0.958)	0.135	0.750	0.827
Test	0.667	0.845	0.824 (0.752 - 0.896)	0.164	0.699	0.775
**RF**						
Training	0.85	0.946	0.970 (0.961 - 0.980)	0.117	0.867	0.914
Validation	0.700	0.874	0.890 (0.834 - 0.947)	0.153	0.709	0.819
Test	0.574	0.833	0.824 (0.754 - 0.894)	0.177	0.626	0.732
**LightGBM**						
Training	0.982	0.952	0.995 (0.992 - 0.998)	0.054	0.944	0.962
Validation	0.725	0.828	0.864 (0.802 - 0.926)	0.140	0.690	0.795
Test	0.667	0.786	0.800 (0.719 - 0.880)	0.178	0.667	0.739
**ET**						
Training	0.62	0.83	0.835 (0.807 - 0.863)	0.197	0.630	0.761
Validation	0.600	0.851	0.845 (0.774 - 0.917)	0.190	0.623	0.772
Test	0.593	0.845	0.781 (0.702 - 0.861)	0.207	0.646	0.746
**XGBoost**						
Training	0.669	0.965	0.940 (0.925 - 0.955)	0.103	0.769	0.868
Validation	0.575	0.920	0.886 (0.825 - 0.947)	0.125	0.657	0.811
Test	0.444	0.905	0.821 (0.751 - 0.892)	0.173	0.558	0.725
**AdaBoost**						
Training	0.445	0.949	0.870 (0.846 - 0.894)	0.236	0.574	0.783
Validation	0.475	0.977	0.882 (0.811 - 0.952)	0.236	0.623	0.819
Test	0.481	0.940	0.825 (0.751 - 0.898)	0.238	0.612	0.761
**GBDT**						
Training	0.761	0.968	0.967 (0.956 - 0.977)	0.078	0.834	0.900
Validation	0.525	0.885	0.872 (0.809 - 0.935)	0.133	0.592	0.772
Test	0.519	0.881	0.835 (0.766 - 0.904)	0.164	0.609	0.739
**SVM**						
Training	0.767	0.831	0.858 (0.832 - 0.885)	0.144	0.727	0.810
Validation	0.800	0.839	0.888 (0.824 - 0.953)	0.126	0.744	0.827
Test	0.685	0.869	0.831 (0.759 - 0.903)	0.164	0.725	0.797
**MLP**						
Training	0.626	0.884	0.858 (0.831 - 0.885)	0.138	0.672	0.799
Validation	0.650	0.920	0.889 (0.826 - 0.952)	0.116	0.712	0.835
Test	0.593	0.881	0.822 (0.749 - 0.895)	0.167	0.667	0.768
**Stacking***						
Training	0.791	0.851	0.900 (0.879 - 0.921)	0.237	0.755	0.831
Validation	0.825	0.851	0.900 (0.842 - 0.959)	0.237	0.767	0.843
Test	0.667	0.845	0.837 (0.769 - 0.906)	0.239	0.699	0.775

The Stacking model was constructed by five base models (RF, LogiR, SVM, MLP and XGBoost) and a logistic regression meta-learner. AUC: area under the curve; LogiR: logistic regression; RF: random forest; LightGBM: light gradient boosting machine; ET: extra trees; XGBoost: extreme gradient boosting; AdaBoost: adaptive boosting; GBDT: gradient boosting decision tree; SVM: Support vector machine; MLP: multilayer perceptron

Table S4. Performance of machine learning-based models for former smokers

	Recall	Specificity	AUC (95%CI)	Brier score	F1	Accuracy
**LogiR**						
Training	0.743	0.780	0.846 (0.829 - 0.864)	0.158	0.68	0.768
Validation	0.649	0.849	0.816 (0.761 - 0.870)	0.156	0.670	0.781
Test	0.698	0.791	0.804 (0.750 - 0.858)	0.177	0.667	0.759
**RF**						
Training	0.810	0.916	0.948 (0.939 - 0.957)	0.134	0.819	0.881
Validation	0.629	0.903	0.837 (0.787 - 0.888)	0.167	0.693	0.809
Test	0.635	0.802	0.800 (0.747 - 0.853)	0.182	0.632	0.745
**LightGBM**						
Training	0.743	0.839	0.873 (0.858 - 0.889)	0.156	0.719	0.807
Validation	0.660	0.876	0.856 (0.808 - 0.903)	0.156	0.696	0.802
Test	0.677	0.769	0.809 (0.757 - 0.861)	0.176	0.640	0.737
**ET**						
Training	0.772	0.813	0.885 (0.870 - 0.900)	0.173	0.719	0.800
Validation	0.660	0.823	0.820 (0.767 - 0.872)	0.187	0.660	0.767
Test	0.635	0.753	0.769 (0.709 - 0.828)	0.200	0.604	0.712
**XGBoost**						
Training	0.651	0.952	0.919 (0.908 - 0.931)	0.113	0.745	0.852
Validation	0.505	0.946	0.849 (0.800 - 0.897)	0.140	0.628	0.795
Test	0.490	0.901	0.811 (0.760 - 0.862)	0.162	0.584	0.759
**AdaBoost**						
Training	0.451	0.947	0.845 (0.828 - 0.863)	0.239	0.579	0.782
Validation	0.412	0.989	0.838 (0.788 - 0.889)	0.238	0.576	0.792
Test	0.469	0.934	0.803 (0.750 - 0.855)	0.240	0.588	0.773
**GBDT**						
Training	0.684	0.961	0.929 (0.918 - 0.940)	0.104	0.776	0.869
Validation	0.536	0.93	0.835 (0.786 - 0.884)	0.145	0.642	0.795
Test	0.490	0.890	0.808 (0.757 - 0.858)	0.165	0.577	0.752
**SVM**						
Training	0.746	0.791	0.846 (0.828 - 0.864)	0.146	0.689	0.776
Validation	0.639	0.871	0.821 (0.768 - 0.874)	0.150	0.678	0.792
Test	0.667	0.786	0.802 (0.748 - 0.857)	0.167	0.643	0.745
**MLP**						
Training	0.676	0.912	0.894 (0.881 - 0.908)	0.119	0.729	0.833
Validation	0.577	0.887	0.817 (0.764 - 0.869)	0.159	0.644	0.781
Test	0.573	0.868	0.799 (0.745 - 0.852)	0.170	0.629	0.766
**Stacking***						
Training	0.796	0.884	0.846 (0.829 - 0.864)	0.228	0.785	0.855

Validation	0.649	0.887	0.816 (0.761 - 0.870)	0.232	0.696	0.806
Test	0.667	0.824	0.804 (0.750 - 0.858)	0.235	0.667	0.770

The Stacking model was constructed by five base models (RF, AdaBoost, GBDT, lightGBM and XGBoost) and a logistic regression meta-learner. AUC: area under the curve; LogiR: logistic regression; RF: random forest; LightGBM: light gradient boosting machine; ET: extra trees; XGBoost: extreme gradient boosting; AdaBoost: adaptive boosting; GBDT: gradient boosting decision tree; SVM: Support vector machine; MLP: multilayer perceptron

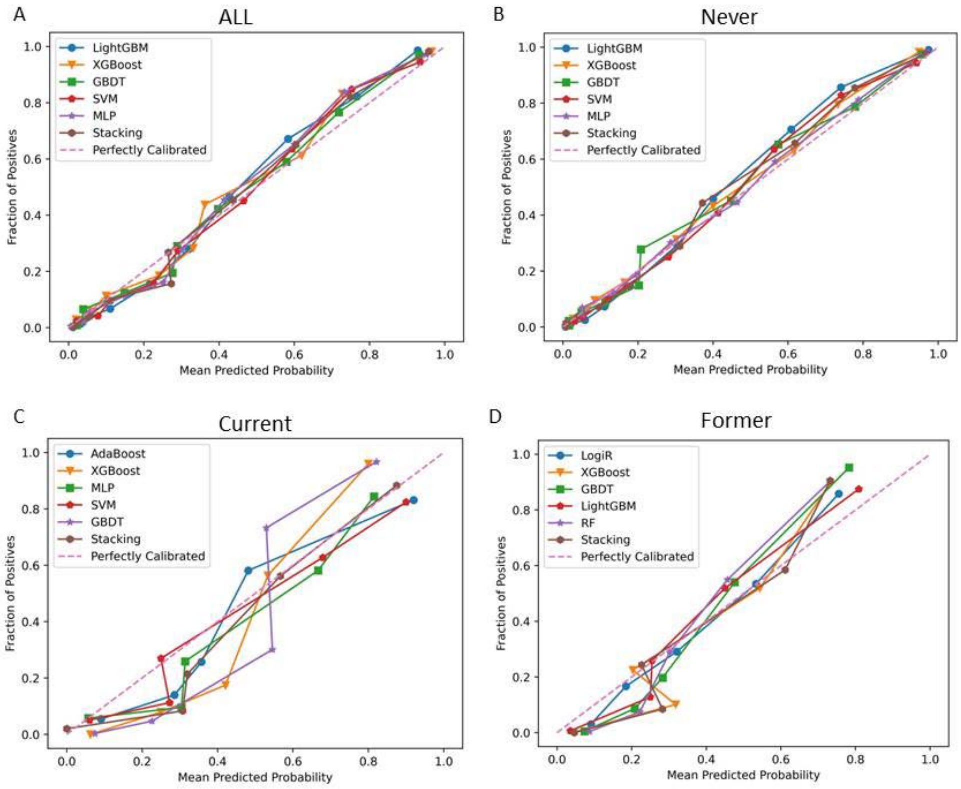

Figure S1. Calibration curves of base and stacking machine learning models for predictions of lung cancer in test dataset. A. CALIBRATION curves of MLP, LightGBM, GBDT, SVM and XGBoost and Stacking model in full data. **B.** CALIBRATION curves of MLP, LightGBM, GBDT, SVM and XGBoost and Stacking model in never smokers. **C.** CALIBRATION curves of AdaBoost, GBDT, SVM, MLP and XGBoost and Stacking model in current smokers. **D.** CALIBRATION curves of LogiR, GBDT, RF, lightGBM and XGBoost and Stacking model in former smokers.
